# Dr. Louis A. Weigel

**Published:** 1906-07

**Authors:** 


Dr. Louis A. Weigel, of Rochester, died at his home May 31, 1906, aged 52 years. He was a graduate of the University of Maryland School of Medicine, 1875, and began practice in Rochester soon afterward. He was a member of the Medical Society of the State of New York, of the Rochester Academy of Medicine, of the Rochester Pathological Society, of the Medical Society of the County of Monroe, and orthopedic surgeon to the Rochester City and St. Mary's Hospitals. He was also consulting orthopedic surgeon to the Craig colony for epileptics at Sonyea, N. Y., and had been professor of orthopedic surgery in Niagara University Medical College, Buffalo.

Dr. Weigel was distinctly a specialist in orthopedic surgery, and had been president of the American Orthopedic Association. He began early to experiment with *x*-rays and finally became an acknowledged authority on the subject. Sadly enough, his zeal in the employment of this agent resulted in disease of his hands rendering amputation of some of his fingers necessary a year or two ago. Later, malignant disease appeared which caused his death. Elsewhere we publish a memorial recently adopted by the Rochester Academy of Medicine, of which he was president at the time of his death.

